# Continuous damage parameter calculation under thermo-mechanical random loading

**DOI:** 10.1016/j.mex.2014.07.004

**Published:** 2014-08-08

**Authors:** Marko Nagode

**Affiliations:** University of Ljubljana, Faculty of Mechanical Engineering, Aškerčeva 6, 1000 Ljubljana, Slovenia

**Keywords:** Damage parameter, Mean stress, Thermo-mechanical fatigue, Low cycle fatigue, Hysteresis operator

## Abstract

The paper presents a method on how the mean stress effect on fatigue damage can be taken into account under an arbitrary low cycle thermo-mechanical loading. From known stress, elastoplastic strain and temperature histories the cycle amplitudes and cycle mean values are extracted and the damage parameter is computed. In contrast to the existing methods the proposed method enables continuous damage parameter computation without the need of waiting for the cycles to close. The limitations of the standardized damage parameters are thus surpassed. The damage parameters derived initially for closed and isothermal cycles assuming that the elastoplastic stress–strain response follows the Masing and memory rules can now be used to take the mean stress effect into account under an arbitrary low cycle thermo-mechanical loading. The method includes:

• stress and elastoplastic strain history transformation into the corresponding amplitude and mean values;
• stress and elastoplastic strain amplitude and mean value transformation into the damage parameter amplitude history;
• damage parameter amplitude history transformation into the damage parameter history.

## Graphical abstract

**Figure:**
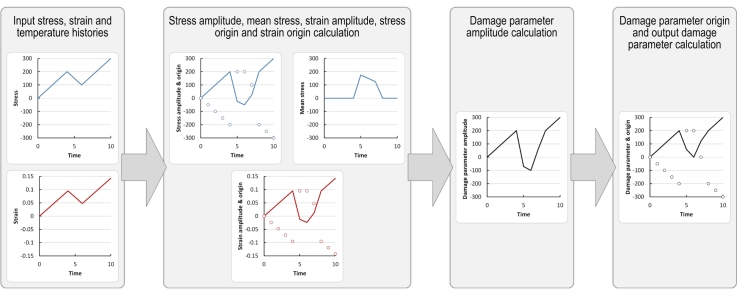

## Method details

### Introduction

It is well known that mean stress affects fatigue life significantly and can therefore not be neglected under low cycle isothermal mechanical loading [1–4]. The mean stress correction formulae [5–7] are applied to transfer the extracted closed cycles with known stress amplitudes *σ*_*a*_, mean stresses *σ*_*m*_ and elastoplastic strain amplitudes $\varepsilon_{a}^{ep}$ into the closed cycles with equivalent damage parameter amplitudes *P*_*a*_. Moreover, closed cycles can be extracted by, e.g., the rainflow counting method [8].

However, under low cycle non-isothermal mechanical loading a cycle closure problem may appear due to variable temperature or strain rate or both, which results in a more difficult determination of the stress and strain amplitudes and mean stresses [3]. The cycle closure problem leads to a more difficult determination of *σ*_*a*_, *σ*_*m*_ and $\varepsilon_{a}^{ep}$ because the cycle counting methods [8] cannot count the cycles before they close. This is particularly important if damage is calculated continuously (at any moment without the need of ‘waiting’ for the cycle to finish), which is the case in the damage operator approach (DOA) [4].

The aim of the method is to extend the usage of damage parameters derived initially for closed cycles and isothermal mechanical loading to arbitrary cycles and non-isothermal mechanical loading.

### Requirements

The stress and strain tensor histories are gained by elastoplastic models from structural finite element analyses (FEA) and are converted into equivalent uniaxial stress *σ*(*t*_*i*_) and equivalent uniaxial elastoplastic strain $\varepsilon^{ep}(t_{i})$ histories for *i* = 1, …, *n*. Thermal FEA are required to assess the corresponding temperature history *T*(*t*_*i*_). Test stand tests can replace FEA. Temperature history *T*(*t*_*i*_) influences the elastoplastic stress–strain response and material parameters, e.g., Young modulus *E*(*t*_*i*_) = *E*(*T*_*i*_), where *T*_*i*_ = *T*(*t*_*i*_) but does not appear in the algorithm directly.

Counter *j* counts the strain reversal points including the first $\varepsilon^{ep}(t_{1})$ and the last $\varepsilon^{ep}(t_{n})$ point in the elastoplastic strain history. The strain reversal points constitute strain residuum $\varepsilon_{j}^{ep,res}$ for j=1, …, ≤n. Current $\varepsilon^{ep}(t_{i})$ and the latest three strain reversal points $\varepsilon_{j}^{ep,res},\;\varepsilon_{j-1}^{ep,res}$, and $\varepsilon_{j-2}^{ep,res}$ are checked for a closed cycle successively. Residuum stresses $\sigma_{j}^{res}$ and residuum damage parameters $P_{j}^{res}$ coincide in time domain with residuum strains $\varepsilon_{j}^{ep,res}$.

Logical operator *s* enables the identification of rainflow cycles that hold nested rainflow cycles. It is set to true if the rainflow cycle is identified and *j* > 2. Otherwise it is false.

### Algorithm flow

**Fig. 1:**
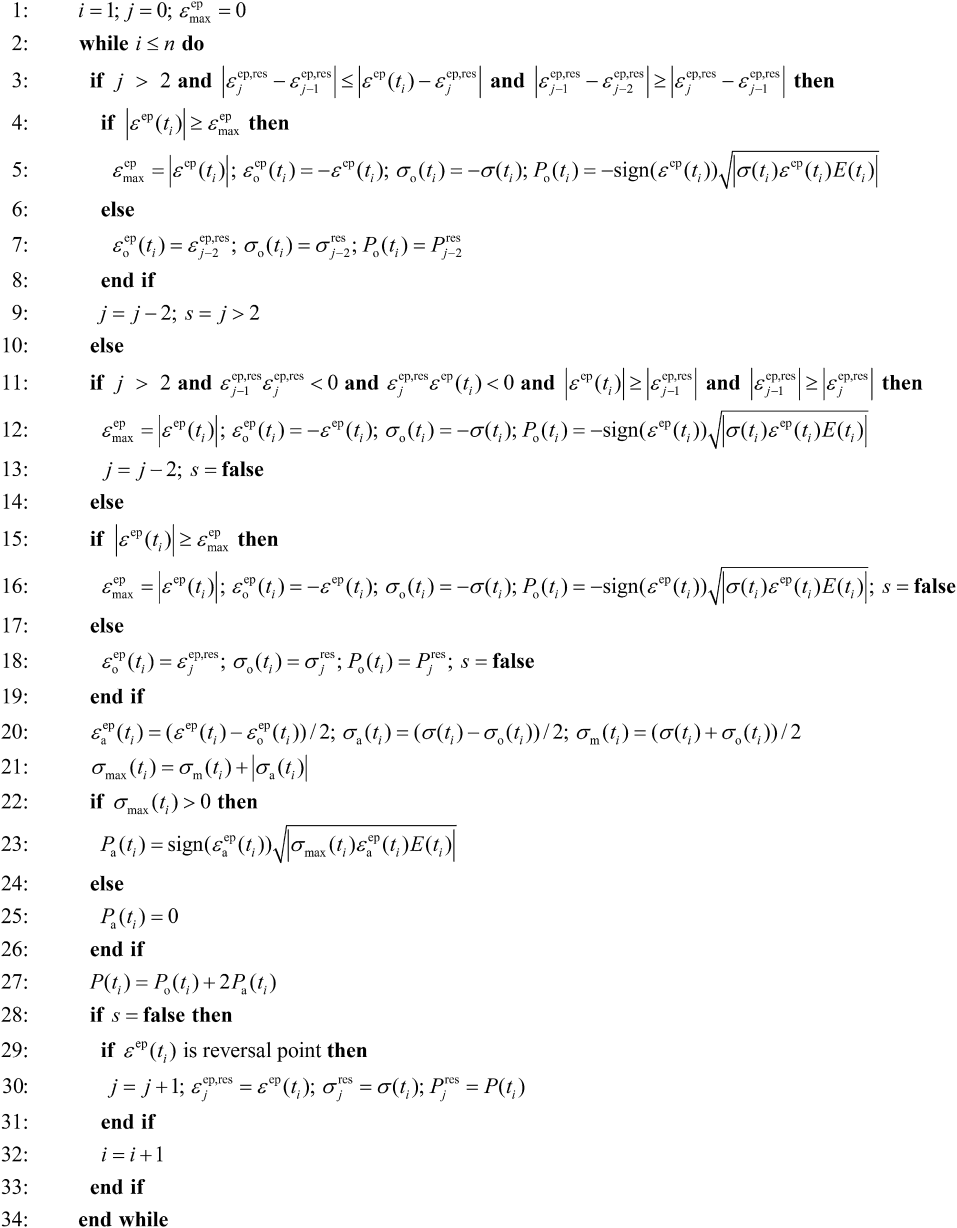
Pseudo code illustrating continuous damage parameter calculation.

The procedure of working out the damage parameter is given in Fig. 1. Here an overview of the pseudo code is provided. In line 1, counters *i*, *j* and maximum absolute strain $\varepsilon_{\max}^{ep}$ are initiated. Superscript *ep* stands for elastoplastic. Next, the outer loop begins. Index *i* runs over *n* available times *t*_*i*_. If the number of strain reversal points in the residuum *j* > 2, the algorithm in line 3 first checks if residuum strains $\varepsilon_{j-2}^{ep,res},\;\varepsilon_{j-1}^{ep,res}$, $\varepsilon_{j}^{ep,res}$ and strain $\varepsilon^{ep}(t_{i})$ form a rainflow [8] cycle.

The three residuum strains representing strain reversal points as well as $\varepsilon^{ep}(t_{i})$ are required for the standardized four point rainflow counting algorithm [8]. The Clormann–Seeger [9] cycle is searched for in line 11. If *j* ≤ 2 or neither rainflow nor Clormann–Seeger cycle is found, the algorithm in line 15 checks if $\varepsilon^{ep}(t_{i})$ is on the cyclic stress–strain curve. Else, strain origin $\varepsilon_{o}^{ep}(t_{i})$, stress origin $\sigma_{o}(t_{i})$ and damage parameter origin $P_{o}(t_{i})$ are set to the *j*-th residuum values and logical operator *s* is set to false in line 18. Origins are required to make the amplitude and mean values calculation possible. They are determined in lines 5, 7, 12, 16 and 18. In line 20 strain amplitude

(1) $$\varepsilon_{a}^{ep}(t_{i})=\frac{\varepsilon^{ep}(t_{i})-\varepsilon_{o}(t_{i})}{2}$$

stress amplitude

(2) $$\sigma_{a}(t_{i})=\frac{\sigma (t_{i})-\sigma_{o}(t_{i})}{2}$$

and mean stress

(3) $$\sigma_{m}(t_{i})=\frac{\sigma (t_{i})+\sigma_{o}(t_{i})}{2}$$

referring to time *t*_*i*_ are calculated. Similarly, mean strain is given by

$$\varepsilon_{m}^{ep}(t_{i})=\frac{\varepsilon^{ep}(t_{i})+\varepsilon_{o}^{ep}(t_{i})}{2}$$

Maximum stress is obtained in line 21 as follows

(4) $$\sigma_{\max}(t_{i})=\sigma_{m}(t_{i})+|\sigma_{a}(t_{i})|$$

If $\sigma_{\max}(t_{i})>0$, damage parameter amplitude in line 23

(5) $$P_{a}(t_{i})=sign(\varepsilon_{a}^{ep}(t_{i}))\sqrt{\left|\sigma_{\max}(t_{i})\varepsilon_{a}^{ep}(t_{i})E(t_{i})\right|}$$

Otherwise, $P_{a}(t_{i})=0$ in line 25. Damage parameter in line 27 referring to time *t*_*i*_ is then

(6) $$P(t_{i})=P_{o}(t_{i})+2P_{a}(t_{i})$$

If *s* in line 28 is false, the algorithm in line 29 checks if $\varepsilon^{ep}(t_{i})$ stands for the strain reversal point. If true, *j* is incremented and residuum strain $\varepsilon_{j}^{ep,res}$, residuum stress $\sigma_{j}^{res}$, residuum damage parameter $P_{j}^{res}$ are set to the *i*-th values.

In lines 4–9 origins $\varepsilon_{o}^{ep}(t_{i}),\sigma_{o}(t_{i})$, and $P_{o}(t_{i})$ are set depending on $\varepsilon^{ep}(t_{i})$ either as in line 16 or to the *j* − 2-th residuum values. Index *j* is decremented and *s* is set to true if *j* > 2. In lines 12–13 origins $\varepsilon_{o}^{ep}(t_{i}),\sigma_{o}(t_{i})$, and $P_{o}(t_{i})$ are set as in line 16. Index *j* is decremented and *s* is set to false. The algorithm can be adapted if SWT expression in lines 5, 12 and 16 is replaced by another damage parameter and if lines 21–26 are adjusted accordingly.

### Example

For simplicity reasons and to enable the point-to-point validation of the pseudo code, the isothermal linear elastic stress–strain response is studied.

**Fig. 2:**
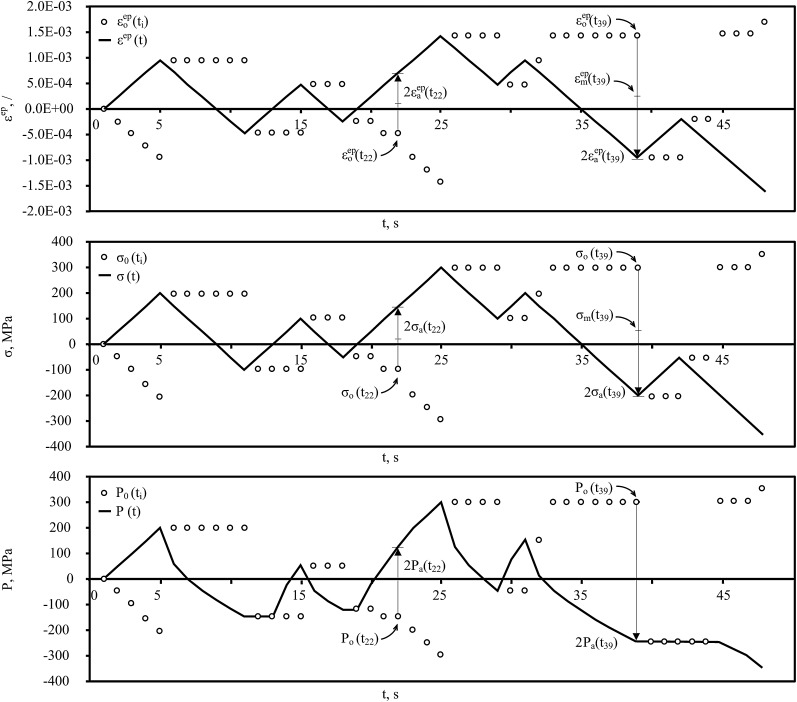
Continuous damage parameter calculation.

Let elastoplastic strain $\varepsilon^{ep}(t_{i})$, stress $\sigma (t_{i})=E\varepsilon^{ep}(t_{i})$, where *E* = 210,000 MPa, and temperature $T(t_{i})=293\;\text{K}$ histories be known (see Fig. 2 and Table 1). From $\varepsilon^{ep}(t_{i})$ history one Clormann–Seeger cycle (strain reversal points for the four point cycle counting algorithm at times *t*_1_, *t*_5_, *t*_11_, *t*_25_) and three rainflow cycles can be identified. The first appears within the Clormann–Seeger cycle (*t*_11_, *t*_15_, *t*_18_, *t*_25_), the second within times *t*_25_, *t*_29_, *t*_31_, *t*_39_ and the third compressive cycle within *t*_31_, *t*_39_, *t*_42_, *t*_48_. The memory [5] M1 rule applies at time *t*_23_, memory M2 rule at *t*_21_, *t*_33_ and at *t*_45_ and memory M3 rule at *t*_47_. As temperature is kept constant in this simple case, the strain, stress and damage parameter reversal points coincide.

Unlike the existing strain life approaches, where amplitude and mean values cannot be determined before cycle closure at times *t*_21_, *t*_23_, *t*_33_ and *t*_45_, the presented algorithm enables the determination of all load parameters at any time *t*_*i*_. It can be noted that the third compressive cycle starting at *t*_39_ results in $P_{a}(t_{41})=P_{a}(t_{42})=P_{a}(t_{43})=P_{a}(t_{44})=0$, which is correct as $P_{a}(t_{i})\neq 0$ only if $\sigma_{\max}(t_{i})>0$ (see line 22 in Fig. 1). The complete calculation is listed in Table 1.

Although damage parameter *P*(*t*_*i*_) is not a monotonic function, it still results in monotonically increasing fatigue damage *D*_*f*_(*t*_*i*_) as it is expressed in the form of *total variation* introduced in Ref. [10]. Since the damage parameter history is now known, fatigue damage *D*_*f*_(*t*_*i*_) can be calculated as explained in detail in Section 5.3 of Ref. [11].

### Results

The strain-life approach is standardized and widely accepted for determining fatigue damage under strain-controlled low cycle fatigue loading. It is a standard practise to use damage parameters to account for the mean stress correction here. However, to perform the conversion of the load parameters into the damage parameter, temperature should be kept constant.

The presented method generalizes the damage parameters by continuous calculation of the stress and strain amplitude and mean stress instead of waiting for the cycles to close. Not only the discussed Smith–Watson–Topper damage parameter but also other parameters [5,7] can now be used identically in the strain-life predictions under thermo-mechanical cyclic loading. The method will enable an application of widely used strain-life mean stress correction techniques in energy based approaches such as in Refs. [12–14].

## Additional information

### Background

Traditionally, after identifying a closed cycle the value of damage parameter is computed. There are several definitions of damage parameters, such as Morrow, Smith–Watson–Topper (SWT) or Nihei [5] that have been derived for closed and isothermal cycles assuming that the elastoplastic stress–strain response follows the Masing [15] and memory [5] rules. In this paper the well-known and widely used Smith–Watson–Topper [6] damage parameter is addressed. Other damage parameters [7] could be considered equally. The SWT damage parameter for a closed cycle is given as follows

(7) $$P_{a}=\sqrt{(\sigma_{a}+\sigma_{m})\varepsilon_{a}^{ep}E}$$

The corresponding SWT damage parameter amplitude at arbitrary times $0\leq t_{1}\leq t_{2}\leq \cdots \leq t_{i}\leq \cdots$ is given by [4]

(8) $$P_{a}(t_{i})=\sqrt{\left(\sigma_{a}(t_{i})+\sigma_{m}(t_{i}))\varepsilon_{a}^{ep}(t_{i})E(T_{i}\right)}$$

where the Young modulus *E*(*T*_*i*_) depends on temperature *T*_*i*_. Eq. (8) represents a damage parameter amplitude requiring continuous information on stress amplitude *σ*_a_(*t*_*i*_), elastoplastic strain amplitude $\varepsilon_{a}^{ep}(t_{i})$ and mean stress *σ*_*m*_(*t*_*i*_). Let us suppose that stress *σ*(*t*_*i*_), elastoplastic strain $\varepsilon^{ep}(t_{i})$ and temperature *T*(*t*_*i*_) histories are known. They either result from the thermo-mechanical finite element analysis or test stand tests.

**Fig. 3:**
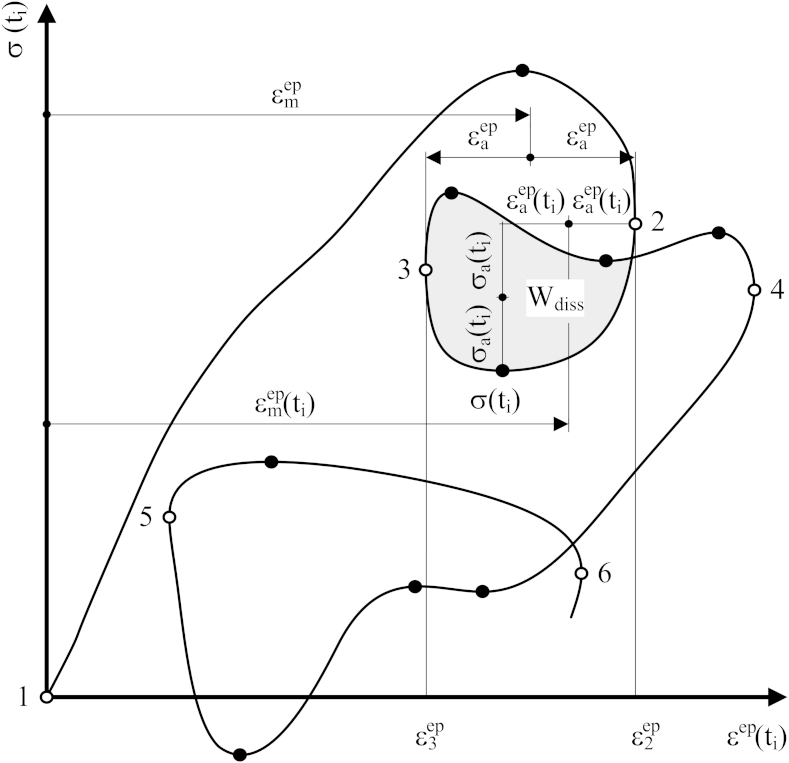
Stress–strain response and dissipated energy.

If temperature dependent cyclically stable cyclic stress–strain curves increase monotonically and if temperature and strain rate are kept constant, stress as a function of strain between the strain reversal points increases or decreases monotonically. Consequently, stress and strain reversal points coincide [5]. An elastoplastic stress–strain response reflecting an arbitrary thermo-mechanical loading is sketched in Fig. 3. The stress reversal points in the black circles and strain reversal points in the white ones do not coincide. This comes from variable temperature.

The existing strain-life approaches have been derived for stress–strain responses with coincidental stress and strain reversal points for which *σ*_*a*_, *σ*_*m*_ and $\varepsilon_{a}^{ep}$ can be determined in a simple and straightforward way [8]. However, for the elastoplastic stress–strain response as depicted in Fig. 3 it has not been possible to uniquely identify the three values yet. If elastoplastic stress–strain response is interpreted in terms of dissipated energy

(9) $$W^{diss}=\int_{cycle}\sigma d\varepsilon^{ep}$$

where the integration boundaries are the strain reversal points

(10) $$W^{diss}=\int_{\varepsilon_{2}^{ep}}^{\varepsilon_{3}^{ep}}\sigma d\varepsilon^{ep}+\int_{\varepsilon_{3}^{ep}}^{\varepsilon_{2}^{ep}}\sigma d\varepsilon^{ep}$$

the problem can be solved. The dissipated energy of the closed cycle in grey between points 2 and 3 in Fig. 3 can obviously be obtained without knowing the stress-reversal points. The corresponding elastoplastic strain amplitude $\varepsilon_{a}^{ep}$ and elastoplastic mean strain $\varepsilon_{m}^{ep}$ can thus be extracted by applying the Masing and memory rules on elastoplastic strain $\varepsilon^{ep}(t_{i})$ history only. Moreover, $\varepsilon^{ep}(t_{i})$ also contains continuous information on elastoplastic strain amplitude $\varepsilon_{a}^{ep}(t_{i})$ and elastoplastic mean strain $\varepsilon_{m}^{ep}(t_{i})$.

## Figures and Tables

**Table 1:** Continuous damage parameter calculation.

*t*_*i*_ (s)	$\varepsilon^{ep}(t_{i})$	*σ*(*t*_*i*_) (MPa)	$\varepsilon_{o}^{ep}(t_{i})$ (–)	$\varepsilon_{a}^{ep}(t_{i})$ (–)	*σ*_*o*_(*t*_*i*_) (MPa)	*σ*_*a*_(*t*_*i*_) (MPa)	*σ*_*m*_(*t*_*i*_) (MPa)	*P*(*t*_*i*_) (MPa)	*P*_*o*_(*t*_*i*_) (MPa)	*P*_*a*_(*t*_*i*_) (MPa)
1	0.000000	0	0.000000	0.000000	0	0	0	0.0	0.0	0.0
2	0.000238	50	−0.000238	0.000238	−50	50	0	50.0	−50.0	50.0
3	0.000476	100	−0.000476	0.000476	−100	100	0	100.0	−100.0	100.0
4	0.000714	150	−0.000714	0.000714	−150	150	0	150.0	−150.0	150.0
5	0.000952	200	−0.000952	0.000952	−200	200	0	200.0	−200.0	200.0
6	0.000714	150	0.000952	−0.000119	200	−25	175	58.6	200.0	−70.7
7	0.000476	100	0.000952	−0.000238	200	−50	150	0.0	200.0	−100.0
8	0.000238	50	0.000952	−0.000357	200	−75	125	−44.9	200.0	−122.5
9	0.000000	0	0.000952	−0.000476	200	−100	100	−82.8	200.0	−141.4
10	−0.000238	−50	0.000952	−0.000595	200	−125	75	−116.2	200.0	−158.1
11	−0.000476	−100	0.000952	−0.000714	200	−150	50	−146.4	200.0	−173.2
12	−0.000238	−50	−0.000476	0.000119	−100	25	−75	−146.4	−146.4	0.0
13	0.000000	0	−0.000476	0.000238	−100	50	−50	−146.4	−146.4	0.0
14	0.000238	50	−0.000476	0.000357	−100	75	−25	−23.9	−146.4	61.2
15	0.000476	100	−0.000476	0.000476	−100	100	0	53.6	−146.4	100.0
16	0.000238	50	0.000476	−0.000119	100	−25	75	−46.4	53.6	−50.0
17	0.000000	0	0.000476	−0.000238	100	−50	50	−87.8	53.6	−70.7
18	−0.000238	−50	0.000476	−0.000357	100	−75	25	−119.6	53.6	−86.6
19	0.000000	0	−0.000238	0.000119	−50	25	−25	−119.6	−119.6	0.0
20	0.000238	50	−0.000238	0.000238	−50	50	0	−19.6	−119.6	50.0
21	0.000476	100	−0.000476	0.000476	−100	100	0	53.6	−146.4	100.0
22	0.000714	150	−0.000476	0.000595	−100	125	25	127.5	−146.4	136.9
23	0.000952	200	−0.000952	0.000952	−200	200	0	200.0	−200.0	200.0
24	0.001190	250	−0.001190	0.001190	−250	250	0	250.0	−250.0	250.0
25	0.001429	300	−0.001429	0.001429	−300	300	0	300.0	−300.0	300.0
26	0.001190	250	0.001429	−0.000119	300	−25	275	126.8	300.0	−86.6
27	0.000952	200	0.001429	−0.000238	300	−50	250	55.1	300.0	−122.5
28	0.000714	150	0.001429	−0.000357	300	−75	225	0.0	300.0	−150.0
29	0.000476	100	0.001429	−0.000476	300	−100	200	−46.4	300.0	−173.2
30	0.000714	150	0.000476	0.000119	100	25	125	76.1	−46.4	61.2
31	0.000952	200	0.000476	0.000238	100	50	150	153.6	−46.4	100.0
32	0.000714	150	0.000952	−0.000119	200	−25	175	12.2	153.6	−70.7
33	0.000476	100	0.001429	−0.000476	300	−100	200	−46.4	300.0	−173.2
34	0.000238	50	0.001429	−0.000595	300	−125	175	−87.3	300.0	−193.6
35	0.000000	0	0.001429	−0.000714	300	−150	150	−124.3	300.0	−212.1
36	−0.000238	−50	0.001429	−0.000833	300	−175	125	−158.3	300.0	−229.1
37	−0.000476	−100	0.001429	−0.000952	300	−200	100	−189.9	300.0	−244.9
38	−0.000714	−150	0.001429	−0.001071	300	−225	75	−219.6	300.0	−259.8
39	−0.000952	−200	0.001429	−0.001190	300	−250	50	−247.7	300.0	−273.9
40	−0.000714	−150	−0.000952	0.000119	−200	25	−175	−247.7	−247.7	0.0
41	−0.000476	−100	−0.000952	0.000238	−200	50	−150	−247.7	−247.7	0.0
42	−0.000238	−50	−0.000952	0.000357	−200	75	−125	−247.7	−247.7	0.0
43	−0.000476	−100	−0.000238	−0.000119	−50	−25	−75	−247.7	−247.7	0.0
44	−0.000714	−150	−0.000238	−0.000238	−50	−50	−100	−247.7	−247.7	0.0
45	−0.000952	−200	0.001429	−0.001190	300	−250	50	−247.7	300.0	−273.9
46	−0.001190	−250	0.001429	−0.001310	300	−275	25	−274.5	300.0	−287.2
47	−0.001429	−300	0.001429	−0.001429	300	−300	0	−300.0	300.0	−300.0
48	−0.001667	−350	0.001667	−0.001667	350	−350	0	−350.0	350.0	−350.0
